# Epidemiology in Action Course

**Published:** 2013-03-08

CDC and Rollins School of Public Health at Emory University will cosponsor the course, Epidemiology in Action, to be held June 3–14, 2013, at Emory University in Atlanta, Georgia. This course is designed for state and local public health professionals.

This course emphasizes practical application of epidemiology to public health problems and consists of lectures, workshops, classroom exercises (including actual epidemiologic problems), and roundtable discussions. Topics scheduled for presentation include descriptive epidemiology and biostatistics, analytic epidemiology, epidemic investigations, public health surveillance, surveys and sampling, and Epi Info training, along with discussions of selected prevalent diseases. Tuition is charged.

Additional information and applications are available at http://www.sph.emory.edu/epicourses ; or by mail (Emory University, Hubert Department of Global Health [Attn: Pia Valeriano], 1518 Clifton Rd. NE, CNR Bldg., Rm. 7038, Atlanta, GA 30322); telephone (404-727-3485); fax (404-727-4590); or e-email ( pvaleri@emory.edu ).

